# Interactive Effects of Vitamin A and All-Trans Retinoic Acid on Growth Performance, Intestinal Health, and Plasma Metabolomics of Broiler Chickens

**DOI:** 10.3390/ani15203005

**Published:** 2025-10-16

**Authors:** Shuangshuang Guo, Yushu Xiong, Lai He, Jiakun Yan, Peng Li, Changwu Li, Binying Ding

**Affiliations:** Hubei Key Laboratory of Animal Nutrition and Feed Science, Wuhan Polytechnic University, Wuhan 430023, China; guo1shuangshuang@163.com (S.G.); xiongyushu0506@gmail.com (Y.X.); hlshasha@163.com (L.H.); yanjk16@whpu.edu.cn (J.Y.); pengli@whpu.edu.cn (P.L.)

**Keywords:** vitamin A, all-trans retinoic acid, intestinal health, metabolomics, broiler chicken

## Abstract

**Simple Summary:**

Vitamin A is an essential lipid-soluble vitamin for broiler chickens. All-trans retinoic acid is one of active metabolites derived from vitamin A and plays a key role in the improvement of intestinal barrier integrity. This study examined the optimal combination of vitamin A and all-trans retinoic acid used in broiler chickens. All-trans retinoic acid and vitamin A increases growth performance of broiler chickens in the starter and grower phases, respectively. High doses of vitamin A and all-trans retinoic acid synergistically improved intestinal health and vitamin A metabolism as well as lipid metabolism in plasma.

**Abstract:**

This study investigated the interactive effects of dietary vitamin A (VA) and all-trans retinoic acid (ATRA) on growth performance and intestinal health in broilers. A total of 432 one-day-old male Arbor Acres chicks were assigned to a 2 × 3 factorial design with two VA levels (2000 and 6000 IU/kg) and three ATRA levels (0, 0.25, and 0.50 mg/kg). The maize–soybean meal basal diet contained 180 IU/kg VA without extra VA supplementation. Results showed that compared with 0 mg/kg ATRA, 0.50 mg/kg ATRA enhanced average daily gain (ADG) during days 1–21 (*p* < 0.05). Compared with 2000 IU/kg VA, 6000 IU/kg VA improved body weight on day 35 as well as ADG and feed intake during days 22–35 and reduced feed conversion ratio over the entire trial (*p* < 0.05). There were VA × ATRA interactions for the ratio of villus height (VH) to crypt depth (CD) in duodenum as well as VH and CD in ileum on day 21 (*p* < 0.05). The 0.25 mg/kg ATRA decreased duodenal VH/CD and ileal VH in broilers fed 2000 and 6000 IU/kg VA, respectively (*p* < 0.05). The 0.50 mg/kg ATRA increased ileal VH in broilers fed both 2000 and 6000 IU/kg VA (*p* < 0.05). When birds were fed 6000 IU/kg VA, 0.50 mg/kg ATRA increased ileal CD compared with 0.25 mg/kg CD (*p* < 0.05). On day 35, compared with 0 mg/kg ATRA, 0.25 mg/kg ATRA increased ileal VH while 0.50 mg/kg ATRA decreased ileal CD, and both of them increased ileal VH/CD (*p* < 0.05). The VA × ATRA interactions for mRNA expression of jejunal *Mucin5ac* on day 21 and jejunal *Occludin*, *Claudin-1*, *Mucin 2*, leucine-rich-repeat-containing G-protein-coupled receptor 5^+^ (*Lgr5^+^*), zinc and ring finger 3 (*Znrf3*), and secreted phosphoprotein 1 (*SPP1*) on day 35 were detected (*p* < 0.05). Dietary 0.50 mg/kg ATRA up-regulated jejunal *Mucin5ac* expression in broilers fed 6000 IU/kg VA on day 21 as well as *Claudin-1*, *Znrf3*, and *SPP1* expression broilers fed 2000 IU/kg VA on day 35 (*p* < 0.05). The 0.25 mg/kg ATRA down-regulated *Occludin* expression in broilers fed 6000 IU/kg VA on day 35 (*p* < 0.05). The 0.25 mg/kg ATRA decreased and increased *Lgr5^+^* expression on day 35 in broilers fed 2000 and 6000 IU/kg VA, respectively (*p* < 0.05). Both 0.25 and 0.50 mg/kg ATRA down-regulated *Mucin-2* expression in broilers fed 2000 IU/kg VA on day 35 (*p* < 0.05). The VA × ATRA interactions were observed for jejunal retinol dehydrogenase 10 (RDH10), cytochrome P450, family 26, subfamily A, polypeptide 1 (*CYP26A1*), retinoic acid receptor (*RAR*) α, and *RARβ* expression on days 21 and 35 (*p* < 0.05). Both 0.25 and 0.50 mg/kg up-regulated *RDH10*, *CYP26A1*, and RARβ expression in broilers fed 6000 IU/kg VA (*p* < 0.05). The *RAR*α expression was up-regulated by 0.50 and 0.25 mg/kg ATRA on days 21 and 35, respectively (*p* < 0.05). Plasma metabolomics identified 269 VA- and 185 ATRA-associated differential metabolites, primarily enriched in lipid metabolism, vitamin digestion and absorption, and bacterial infection pathways. In conclusion, dietary 0.50 mg/kg ATRA and 6000 IU/kg VA enhanced growth performance, intestinal integrity, and VA metabolism, partly through activation of retinoic acid receptors and modulation of plasma lipid metabolism.

## 1. Introduction

Vitamin A (VA) is an essential nutrient in poultry diets and plays a crucial role in promoting growth, maintaining immune function, and ensuring overall health. The VA supplementation in broiler diet has been shown to improve body weight gain up to 42 days [1]. The standard diet (including 10,000 IU/kg of VA) fortified with 6000 IU/kg of VA in the laying hens enhanced reproductive efficiency, antioxidant capacity, and immunity but reduced serum triglyceride concentration [2]. Moreover, oral VA supplementation in specific pathogen-free chickens lowered the mRNA expression levels of some cytokines (interferon-gamma, interleukin (IL)-1β, and IL-6), which served as biomarkers of inflammatory stress [3]. The VA deficiency impairs development of the small intestine of chickens [4]. Conversely, excess VA causes toxicity, also known as hypervitaminosis A, leading to hepatocyte injury, necrosis, stellate cell hyperplasia, and subsequent fibrosis [5,6]. Furthermore, pathogenic infection, including coccidia and *Clostridium perfringens*, impairs poultry production and gut injury via damage to intestinal barrier function and immune responses. These detrimental effects, however, can be alleviated by dietary VA supplementation [7,8].

The VA exists in the body in multiple forms, including retinol, retinal, and retinoic acid, which mediate its diverse biological functions [9]. Firstly, dietary VA is absorbed as retinol. Subsequently, retinol binds to retinol-binding protein (RBP4) and transthyretin in the blood. Retinol and retinoic acid enter cells via membrane diffusion or membrane transport carriers. Retinol binds to cellular retinol-binding protein (CRBP) and is metabolized into retinoic acid via retinal. Retinol is converted to retinoic acid by a two-step reaction in the cytoplasm of intestinal epithelial cells and immune cells. The first step is a reversible reaction, in which retinol is converted into retinal under the catalysis of retinol dehydrogenase (RDH). However, the second step is an irreversible reaction, and retinal can be changed into three forms (all-trans retinoic acid (ATRA), 9-cis retinoic acid, and 13-cis retinoic acid) under the key enzyme of retinal dehydrogenase (RALDH). Among these forms, ATRA exhibits the highest biological activity and physiological relevance. Once synthesized, retinoic acid is transported to the nucleus by cellular retinoic acid-binding protein (CRABP) or fatty acid-binding protein 5 (FABP5). Retinoic acid transported by CRABP can bind to retinoic acid receptor (RAR) or retinoid receptor X (RXR) in the nucleus, and the RAR/RXR heterodimer binds to DNA retinoic acid-responsive elements (RAREs) to regulate the expression of genes related to cell growth, apoptosis, and immune response [10].

The potent biological effects of ATRA have been demonstrated in various species. For instance, in ovo injection of ATRA promoted adipose deposition with hypertrophy during embryonic development, but its effects were not maintained in early posthatch age in broiler chickens [11]. In weaned piglets, ATRA can alleviate transmissible gastroenteritis virus-induced growth inhibition, intestinal barrier damage and intestinal inflammatory response [12]. Despite these promising findings, direct evidence regarding the effects of ATRA on the growth performance, intestinal barrier function, and metabolic mechanisms in broiler chickens remains limited. This study aims to explore whether the combined supplementation of VA and ATRA in diets can affect growth performance, intestinal integrity, and plasma metabolism of broiler chickens, providing novel insights into their potential synergistic effects in poultry production.

## 2. Materials and Methods

### 2.1. Experimental Animals, Diets, and Design

All experimental procedures were approved by the Institutional Animal Care and Use Committee of Wuhan Polytechnic University (WPU202304002). A 2 × 3 factorial design was applied to evaluate the interactive effects of dietary VA and ATRA on the growth performance and intestinal health of broilers. Two levels of VA (2000 and 6000 IU/kg) and three levels of ATRA (0, 0.25, and 0.50 mg/kg) were tested. The 2000 and 6000 IU/kg were inadequate and adequate VA levels for growth performance of broilers [13]. The 0.25 and 0.50 mg/kg ATRA is not toxic for broilers [14]. A total of 432 one-day-old male Arbor Acres broilers (Xiangyang Charoen Pokphand Co., Ltd., Xiangyang, China) were randomly divided into six treatment groups, each with six replicates of 12 birds. The basal diet without extra VA supplementation was a maize–soybean meal formulation that met or approached China Feeding Standard of Chicken (NY/T 33-2004) [15]. Vitamin A contents in basal starter and grower diets were 158 and 174 IU/kg, and β-carotene contents were 8.2 and 10.4 mg/kg. The chicks did not receive VA in ovo or perinatal exposures. The trial lasted 35 days and was divided into starter (days 1–21) and grower (days 22–35) periods. Birds were allowed ad libitum access to feed and water. The ingredient composition and nutrient levels of the basal diet are shown in Table 1. Retinyl acetate (providing 500,000 IU/g of VA) and ATRA (≥98%) were supplied by Beijing Blooming Bio-tech Co., Ltd. (Beijing, China) and Sigma-Aldrich (St. Louis, MO, USA) (R2625), respectively.

### 2.2. Growth Performance

At days 1, 21, and 35, after an 8 h fasting period, body weight (BW), and feed intake were measured for each replicate. Average daily gain (ADG), average daily feed intake (ADFI), and feed conversion ratio (FCR) were calculated for the starter (days 1–21), grower (days 22–35), and entire (days 1–35) experimental periods.

### 2.3. Sample Collection

On days 21 and 35, two birds from each replicate were randomly selected. Blood samples were collected from the wing vein using heparin sodium to inhibit coagulation, followed by euthanasia by cervical dislocation. Plasma was obtained by centrifugation at 3000× *g* for 15 min at 4 °C and stored at −80 °C until further analysis. Approximately 1 cm segments of the mid-duodenum, mid-jejunum, and mid-ileum were excised, and digesta was gently flushed. Intestinal segments were fixed in 4% paraformaldehyde and embedded in paraffin. In addition, another 10 cm segment of mid-jejunum were sampled and longitudinally opened. The digesta was gently flushed, and mucosa was scraped. Jejunal mucosa was frozen in liquid nitrogen and stored at −80 °C for RNA extraction.

### 2.4. Intestinal Morphology

Intestinal tissues were sectioned (5 μm thickness), stained with hematoxylin and eosin, and examined under an Olympus BX-41TF microscope with image analysis software (Olympus Corporation, Tokyo, Japan). Villus height (VH) and crypt depth (CD) were blinded determined according to previous research [17]. Ten intact villi from each section were randomly selected for measurement, and the average values for each section were calculated. The VH was defined as the distance from villus tip to crypt opening, and CD as the depth from crypt opening to base. The villus height-to-crypt depth ratio (VH/CD) was then calculated.

### 2.5. Quantitative Real-Time PCR

Total RNA from jejunal mucosa was extracted using TRIzol™ reagent (Invitrogen Life Technologies, Carlsbad, CA, USA) following the manufacturer’s instructions. RNA concentration and purity (OD_260/280_) were assessed with a NanoDrop^®^ ND-2000 spectrophotometer (Thermo Fisher Scientific Inc., Waltham, MA, USA). First-strand cDNA was synthesized using the PrimeScript^®^ RT reagent kit (Takara Bio Inc., Dalian, China). Quantitative PCR was performed on an ABI 7500 Real-Time PCR System (Thermo Fisher Scientific). Relative gene expression was calculated using the 2^−ΔΔCt^ method with *β-actin* as the reference gene. The stability of *β-actin* as reference gene was assessed using delta Ct method and GeNorm analysis, as Wei et al. (2024) [18] described. Primers were synthesized by Sangon Biotech Co., Ltd. (Shanghai, China), and primer sequences are listed in Table 2.

### 2.6. Plasma Metabolomics Analysis

Based on superior beneficial effects of 0.50 mg/kg ATRA on ADG during days 1–21 and intestinal health to 0.25 mg/kg ATRA, plasma samples from six birds on day 35 in four treatments (VA 2000 IU/kg and 6000 IU/kg with or without 0.50 mg/kg ATRA) were used for metabolomic profiling. Analyses were performed by MetWare Biotechnology Co., Ltd. (Wuhan, China) according to previous research [19,20]. Thaw the samples on ice, whirl for around 10 s, and then centrifuge it with 3000× *g* at 4 °C for 5 min. Take 50 μL of one sample and homogenize it with 300 μL of mixture (include acetonitrile, methanol, and internal standard mixture). Whirl the mixture for 3 min. Then, centrifuge it with 12,000× *g* at 4 °C for 10 min. Extract 200 μL of supernatant and incubate it at −20 °C for 30 min. Centrifuge it with 12,000× *g* at 4 °C for 3 min and extract 180 μL of supernatant for further analysis.

The sample extracts were analyzed using a UPLC-ESI-MS/MS system (UPLC, SCIEX, Framingham, MA, USA; MS, Applied Biosystems, Foster, CA, USA). The analytical conditions were as follows: the UPLC column was a Waters ACQUITY Premier HSS T3 (1.8 μm, 2.1 mm × 100 mm, Agilent, Shanghai, China). The solvents were water with 0.1% formic acid (solvent A) and acetonitrile with 0.1% formic acid (solvent B). Sample measurements were conducted using a gradient program with the following conditions: 95% A and 5% B condition. Over 10 min, a linear gradient to 5% A and 95% B was applied resulting in a composition of 5% A and 95% B. This composition was maintained for 1 min. Subsequently, within 1.1 min, a composition of 95% A and 5.0% B was established and maintained for 2.9 min. The parameters were as follows: the flow rate was 0.40 mL per minute, the column oven was maintained at 40 °C, and the injection volume was 4 μL. The effluent was alternatively connected to an ESI-triple quadrupole linear ion trap (QTRAP)-MS. The operating parameters were as follows: source temperature 550 °C; ion spray voltage 5500 V (positive ion mode)/−4000 V (negative ion mode); ion source gas I, gas II, and curtain gas was set at 50, 60, and 35 psi, respectively; and collision-activated dissociation was high. QQQ scans were acquired as multiple reaction monitoring (MRM) experiments with the collision gas (nitrogen) set to 5 psi. Further optimization is employed to achieve the declustering potential and collision energy for individual MRM transitions. A specific set of MRM transitions was monitored for each elution period according to the metabolites present. Each sample analysis was conducted by both positive and negative ion modes. For the quality control (QC) of metabolomic analysis, 10 μL of each sample was pipetted to pool a QC sample. When running sample sets on a column, one QC sample was injected after 10 samples in the sequence. The annotation levels (from 1 to 4) of MSI were employed.

### 2.7. Statistical Analysis

Data were analyzed with SPSS Statistics 27.0 (IBM, Chicago, IL, USA). Data distribution and homogeneity of variances were verified using the Shapiro–Wilk and Levene’s tests, respectively. All the data had an appropriate normal distribution and homogeneity of variances. Two-way ANOVA was conducted to determine the significance of main effects of VA and ATRA as well as their interactions, followed by Duncan’s multiple range test. The mortality was analyzed by Chi-square test. Results were expressed as means with pooled standard error of the mean (SEM). A *p*-value < 0.05 was considered statistically significant.

For plasma metabolomics analysis, principal component analysis (PCA) was conducted using R software (version 4.2.2) (www.r-project.org) to visualize group separation. Orthogonal partial least squares discriminant analysis (OPLS-DA) and Student’s *t*-test were applied to identify differential metabolites. Metabolites were considered significant based on fold change (≥2 or ≤0.5), *p* < 0.05, and VIP ≥ 1. Pathway enrichment was performed using the Human Metabolome Database [21] and Kyoto Encyclopedia of Genes and Genomes (KEGG) Databases [22].

## 3. Results

### 3.1. Growth Performance

The effects of dietary VA and ATRA supplementation on growth performance of broilers were shown in Table 3. Compared with 0 mg/kg ATRA, the addition of 0.50 mg/kg ATRA tended to increase BW at day 21 (*p* = 0.071) and significantly increased ADG from 1 to 21 days of age (*p* < 0.05). Compared with 2000 IU/kg VA, 6000 IU/kg VA elevated BW on day 35 and ADG during days 22–35 and days 1–35 as well as ADFI during days 22–35, and decreased FCR from day 1 to 35 (*p* < 0.05). It also had the tendency to decrease FCR during days 22–35 (*p* = 0.057) and increase ADFI during days 1–35 (*p* = 0.070) as well as mortality during days 1–21 (*p* = 0.055). There were no dead birds during days 22–42. No significant VA × ATRA interaction was detected for growth performance of broilers (*p* > 0.05).

### 3.2. Intestinal Morphology

The effects of VA and ATRA on the intestinal morphology of broiler chickens on day 21 are shown in Table 4. There were significant VA × ATRA interactions on duodenal VH/CD, ileal VH, and CD (*p* < 0.05). When broilers were fed 2000 IU/kg VA, 0.25 mg/kg ATRA decreased duodenal VH/CD (*p* < 0.05), and this effect disappeared in broilers fed 6000 IU/kg VA. When birds were fed 2000 IU/kg VA, ATRA increased ileal VH in a dose-dependent manner; while birds were fed 6000 IU/kg VA, 0.25 mg/kg ATRA decreased ileal VH, and 0.50 mg/kg ATRA increased it (*p* < 0.05). The ATRA did not significantly affect ileal CD in broilers fed 2000 IU/kg VA, but 0.50 mg/kg ATRA had higher ileal CD than that of 0.25 mg/kg ATRA in broilers fed 6000 IU/kg VA (*p* < 0.05). Compared with 2000 IU/kg VA, 6000 IU/kg VA significantly reduced duodenal CD (*p* < 0.05). Compared with 0 and 0.25 mg/kg ATRA, 0.50 mg/kg ATRA greatly increased ileal VH/CD (*p* < 0.05).

On day 35, no VA × ATRA interactions on intestinal morphological traits were observed (*p* > 0.05, Table 5). The 0.25 mg/kg ATRA had higher CD and lower VH/CD than those of 0.5 mg/kg of ATRA (*p* < 0.05). In ileum, compared with 0 mg/kg ATRA, 0.25 mg/kg ATRA increased VH, 0.50 mg/kg ATRA decreased CD, and both of them increased VH/CD (*p* < 0.05).

### 3.3. Gene Expression of Tight Junctions and Mucins in Jejunum

**Figure 1:**
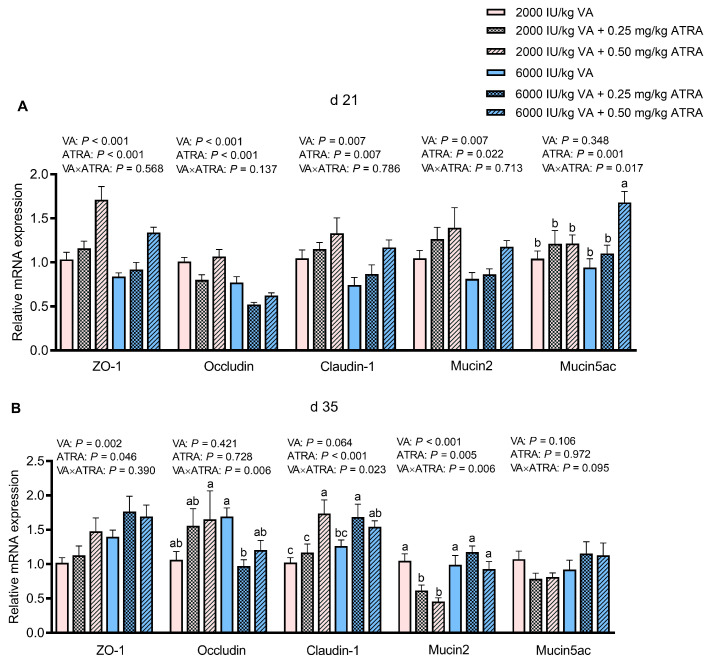
The effects of dietary VA and ATRA supplementation on mRNA expressions of tight junctions and mucins in jejunal mucosa of broiler chickens on days 21 (**A**) and 35 (**B**). Each bar represents the means and standard errors of 12 broilers (*n* = 12). Abbreviations: VA, vitamin A; ATRA, all-trans retinoic acid; ZO-1, Zonula occludens-1. ^a–c^ Bars with different letters differ with significant VA × ATRA interactions (*p* < 0.05).

As exhibited in Figure 1A, compared with 2000 IU/kg VA, 6000 IU/kg VA significantly decreased the relative mRNA expression of *Zonula occludens-1* (*ZO-1*), *Occludin*, *Claudin-1*, and *Mucin2* in jejunum (*p* < 0.05). Compared with 0 and 0.25 mg/kg ATRA, 0.50 mg/kg ATRA significantly up-regulated *ZO-1*, *Claudin-1*, and *Mucin2* expression (*p* < 0.05). The 0.25 mg/kg ATRA had lower *Occludin* expression than that of the other two doses (*p* < 0.05). The VA × ATRA interaction for *Mucin5ac* expression was detected; 0.50 mg/kg ATRA up-regulated *Mucin5ac* expression in broilers fed 6000 IU/kg VA (*p* < 0.05).

As seen in Figure 1B, a large dose of VA (6000 IU/kg) or ATRA (0.50 mg/kg) up-regulated *ZO-1* expression in jejunum on day 35 (*p* < 0.05). The VA × ATRA interaction was observed for *Occludin*, *Claudin-1*, and *Mucin2* expression (*p* < 0.05). The ATRA did not significantly influence *Occludin* expression of broilers fed 6000 IU/kg VA, and 0.25 mg/kg ATRA down-regulated it in broilers fed 2000 IU/kg VA (*p* < 0.05). The 0.50 and 0.25 mg/kg ATRA up-regulated *Claudin-1* expression in broilers fed 2000 and 6000 IU/kg VA, respectively (*p* < 0.05). The 0.25 and 0.50 mg/kg ATRA down-regulated *Mucin2* expression in broilers fed 2000 IU/kg VA (*p* < 0.05) and did not affect it in broilers fed 6000 IU/kg VA.

### 3.4. Gene Expression of Markers Indicating Stem Cell Proliferation in Jejunum

**Figure 2:**
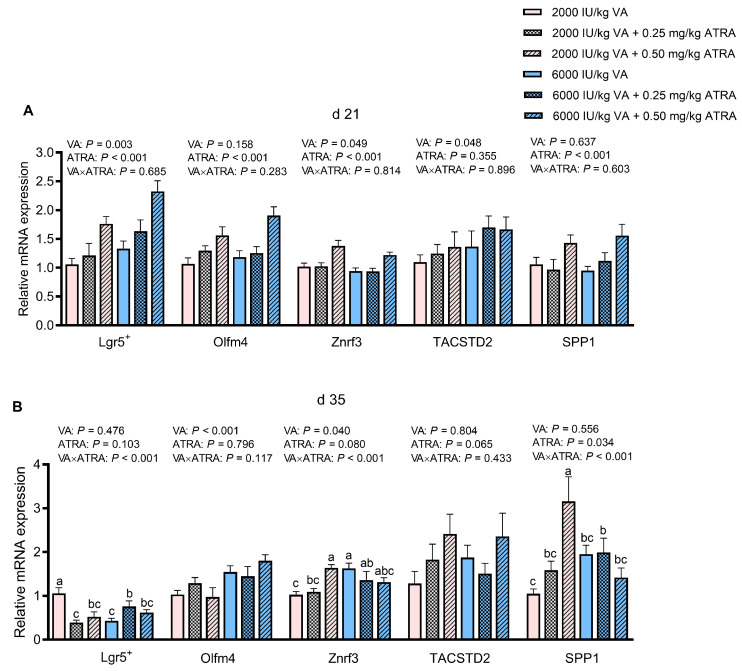
The effects of dietary VA and ATRA supplementation on mRNA expressions of genes involving in stem cell proliferation in jejunal mucosa of broiler chickens on days 21 (**A**) and 35 (**B**). Each bar represents the means and standard errors of 12 broilers (*n* = 12). Abbreviations: VA, vitamin A; ATRA, all-trans retinoic acid; Lgr5^+^, leucine-rich-repeat-containing G-protein-coupled receptor 5^+^; Olfm4, Olfactomedin 4; Znrf3, Zinc and ring finger 3; TACSTD2, Tumor associated calcium signal transducer 2; SPP1, secreted phosphoprotein 1. ^a–c^ Bars with different letters differ with significant VA × ATRA interactions (*p* < 0.05).

As shown in Figure 2A, compared with 2000 IU/kg, 6000 IU/kg VA up-regulated *Leucine-rich-repeat-containing G-protein-coupled receptor 5+ (Lgr5+)*, *Zinc and ring finger 3 (Znrf3)* and *Tumor associated calcium signal transducer 2 (TACSTD2)* expression in jejunum on day 21 (*p* < 0.05). Compared with 0 and 0.25 mg/kg ATRA, 0.50 mg/kg ATRA significantly increased *Lgr5+*, *Olfm4*, *Znrf3*, and *Secreted phosphoprotein 1 (SPP1)* expression (*p* < 0.05). No VA × ATRA interaction was seen for the expression of genes involving in stem cell proliferation (*p* > 0.05).

On day 35, VA × ATRA interactions for the expression of *Lgr5+*, *Znrf3*, and *SPP1* was observed (*p* < 0.05, Figure 2B). The 0.25 and 0.50 mg/kg ATRA down-regulated *Lgr5+* expression in broilers fed 2000 IU/kg VA (*p* < 0.05), while 0.25 mg/kg ATRA up-regulated it in broilers fed 6000 IU/kg VA (*p* < 0.05). The 0.50 mg/kg up-regulated *Znrf3* and *SPP1* expression in broilers fed 2000 IU/kg VA (*p* < 0.05), and this effect disappeared in broilers fed 6000 IU/kg VA. Furthermore, compared with 2000 IU/kg VA, 6000 IU/kg VA up-regulated the *Olfm4* expression (*p* < 0.05).

### 3.5. Gene Expression of Enzymes Involving in Retinoic Acid Metabolism

**Figure 3:**
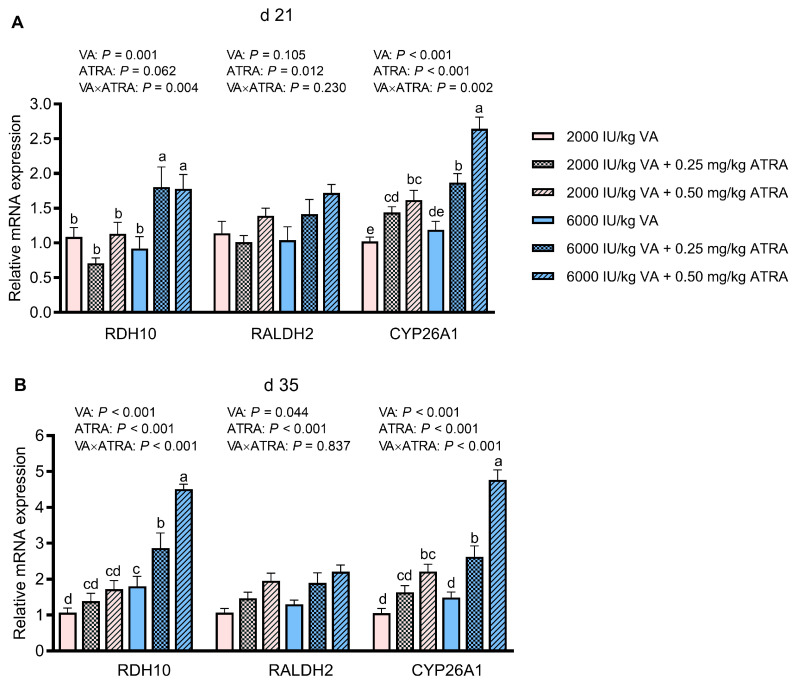
The effects of dietary VA and ATRA supplementation on mRNA expressions of genes associated with retinoic acid metabolism in jejunal mucosa of broiler chickens on days 21 (**A**) and 35 (**B**). Each bar represents the means and standard errors of 12 broilers (*n* = 12). Abbreviations: VA, vitamin A; ATRA, all-trans retinoic acid; RDH, retinol dehydrogenase; RALDH, Retinal dehydrogenase; CYP26A1, cytochrome P450, family 26, subfamily A, polypeptide 1. ^a–e^ Bars with different letters differ with significant VA × ATRA interactions (*p* < 0.05).

The impact of dietary VA and ATRA on mRNA abundance of genes related to retinoic acid metabolism in jejunum is shown in Figure 3A,B. The VA × ATRA interactions were detected for *RDH10* and *Cytochrome P450*, *family 26*, *subfamily A*, *polypeptide 1* (*CYP26A1*) expression at 21 and 35 days of age (*p* < 0.05). The ATRA did not affect *RDH10* mRNA abundance in broilers fed 2000 IU/kg VA on days 21 and 35, but 0.25 and 0.50 mg/kg ATRA up-regulated *RDH10* expression in broilers fed 6000 IU/kg VA (*p* < 0.05). Both 0.25 and 0.50 mg/kg ATRA up-regulated *CYP26A1* expression in broilers fed 2000 IU/kg VA on days 21 and 35 and further elevated it in broilers fed 6000 IU/kg VA (*p* < 0.05). Addition of 0.5 mg/kg ATRA up-regulated the mRNA level of *RALDH2* on days 21 and 35 (*p* < 0.05). Compared with 2000 IU/kg VA, 6000 IU/kg VA increased gene expression of *RALDH2* on day 35 (*p* < 0.05).

### 3.6. Gene Expression of Retinoic Acid Receptors and Retinoic Acid X Receptors

**Figure 4:**
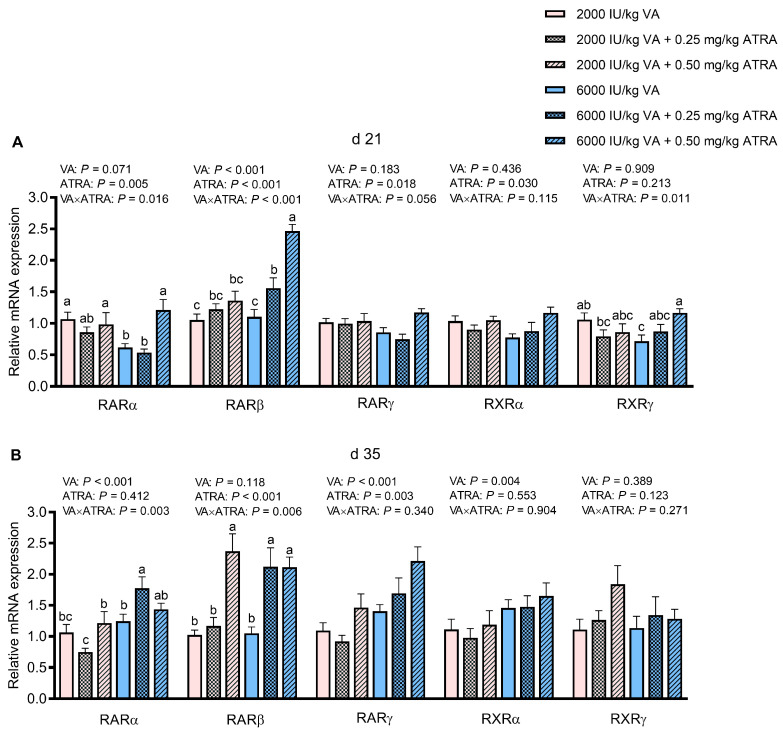
The effects of dietary VA and ATRA supplementation on expression of the genes related to retinoic acid receptors and retinoic acid X receptors in jejunal mucosa of broiler chickens on days 21 (**A**) and 35 (**B**). Each bar represents the means and standard errors of 12 broilers (*n* = 12). Abbreviations: VA, vitamin A; ATRA, all-trans retinoic acid; RAR, retinoic acid receptor; RXR, retinoic acid X receptor. ^a–c^ Bars with different letters differ with significant VA × ATRA interactions (*p* < 0.05).

As shown in Figure 4A,B, VA × ATRA interactions were observed for retinoic acid receptors *RARα* and *RARβ* expression in jejunum on days 21 and 35 as well as *RXRγ* on day 21 (*p* < 0.05). The ATRA did not influence the *RARα*, *RARβ*, and *RXRγ* in birds fed 2000 IU/kg VA on day 21, but 0.50 mg/kg of ATRA up-regulated them in counterparts fed 6000 IU/kg VA (*p* < 0.05). The 0.25 mg/kg of ATRA down-regulated and up-regulated *RARα* expression in broilers fed 2000 and 6000 IU/kg VA on day 35, respectively (*p* < 0.05). The 0.50 mg/kg ATRA and both doses of ATRA up-regulated *RARβ* expression in birds fed 2000 and 6000 IU/kg VA on day 35, respectively (*p* < 0.05). The 0.50 mg/kg ATRA significantly increased *RARγ* and *RXRα* expression on day 21 (*p* < 0.05). Compared with 2000 IU/kg VA, 6000 IU/kg VA up-regulated RARγ and *RXRα* expression on day 35 (*p* < 0.05). In contrast to 0 and 0.25 mg/kg ATRA, 0.50 mg/kg ATRA significantly increased *RARγ* expression on day 35 (*p* < 0.05).

### 3.7. Plasma Metabolome Analysis

**Figure 5:**
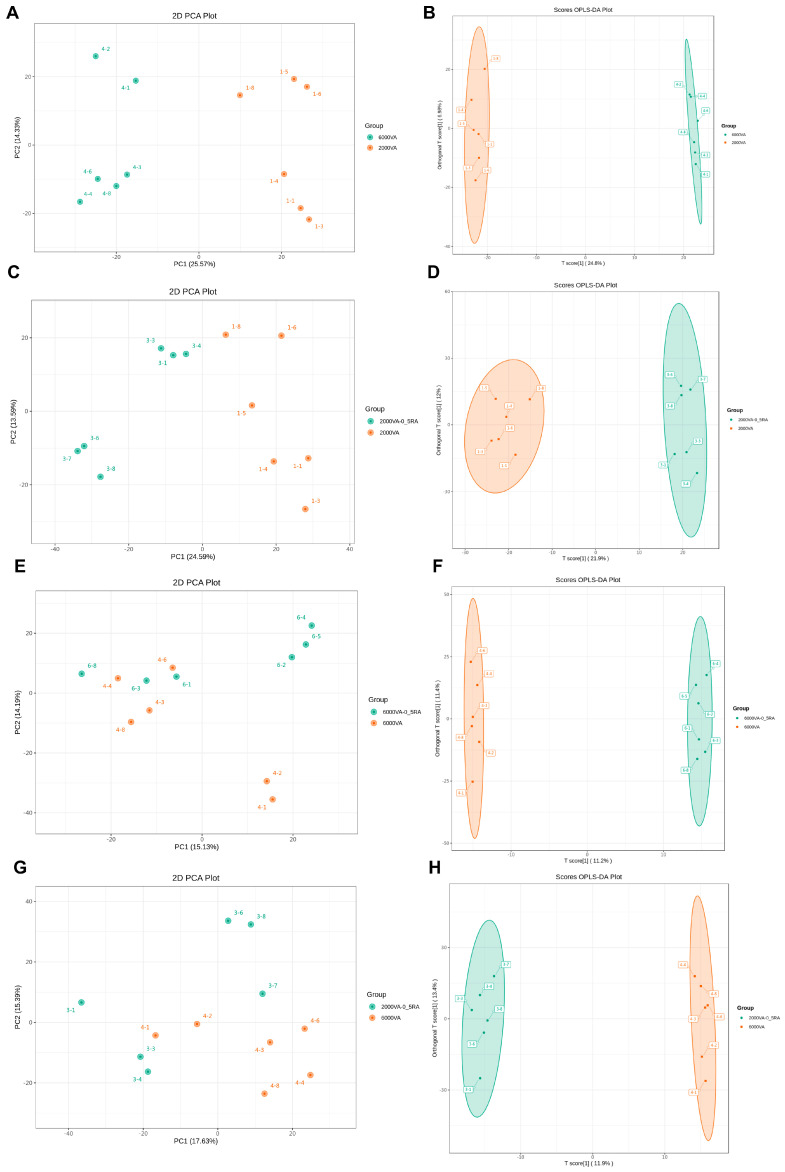
PCA and OPLS-DA analysis of plasma metabolites in broiler chickens on day 35. (**A**,**B**) Metabolic profile of the 6000 IU/kg VA vs. 2000 IU/kg VA group; (**C**,**D**) 2000 IU/kg VA plus 0.5 mg/kg ATRA vs. 2000 IU/kg VA group; (**E**,**F**) 6000 IU/kg VA plus 0.5 mg/kg ATRA vs. 6000 IU/kg VA group; (**G**,**H**) 2000 IU/kg VA plus 0.5 mg/kg ATRA vs. 6000 IU/kg VA group. (**A**,**C**,**E**,**G**) show PCA analysis. (**B**,**D**,**F**,**H**) show OPLS-DA analysis. Abbreviations: VA, vitamin A; ATRA, all-trans retinoic acid.

To delve into the metabolic pathways associated with VA and ATRA, we performed the LC-MS/MS-based metabolomics method to measure differentially enriched metabolites in the plasma. Compared with 0.25 mg/kg ATRA, 0.5 mg/kg ATRA showed superior effects in improving growth performance and intestinal barrier function, as well as stronger modulation of the RAR/RXR signaling pathway. Therefore, 2000 and 6000 IU/kg VA alone or combined with 0.50 mg/kg ATRA were selected for plasma metabolomic analysis. The PCA analysis (Figure 5A,C,E,G) showed that metabolomic profiles were clearly separated between 2000 and 6000 IU/kg VA as well as 2000 IU/kg VA with and without 0.50 mg/kg ATRA. The OPLS-DA analysis (Figure 5B,D,F,H) revealed that plasma samples were well distinguished by different levels of VA and ATRA.

**Figure 6:**
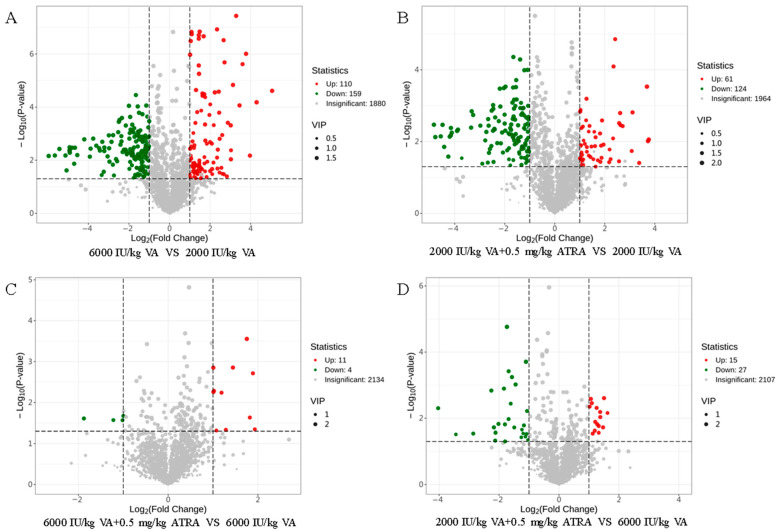
Differential metabolic profiles of plasma between treatment groups. (**A**) The volcano plot of differential metabolites between 6000 IU/kg VA and 2000 IU/kg VA group. (**B**) The volcano plot of differential metabolites between 2000 IU/kg VA plus 0.5 mg/kg ATRA and 2000 IU/kg VA group. (**C**) The volcano plot of differential metabolites between 6000 IU/kg VA plus 0.5 mg/kg ATRA and 6000 IU/kg VA group. (**D**) The volcano plot of differential metabolites between 2000 IU/kg VA plus 0.5 mg/kg ATRA and 6000 IU/kg VA group. Green dots indicate down-regulated metabolites, red dots indicate up-regulated metabolites, and gray dots indicate non-different metabolites.

Differential metabolites between dietary VA and ATRA treatments shown in the form of volcanic plots (Figure 6). A total of 110 up-regulated plasma metabolites and 159 down-regulated metabolites derived from the 6000 IU/kg VA group compared to the 2000 IU/kg VA group (Figure 6A). There were 2149 metabolites and 185 differentially enriched metabolites between the 2000 IU/kg VA group and 2000 IU/kg VA plus 0.5 mg/kg ATRA group, of which 61 were up-regulated and 124 were down-regulated (Figure 6B). When VA level is 6000 IU/kg, 11 metabolites were up-regulated, and 4 metabolites were down-regulated by 0.5 mg/kg ATRA (Figure 6C). Compared with the 6000 IU/kg VA group, 15 metabolites were up-regulated and 27 metabolites were down-regulated in the 6000 IU/kg VA plus 0.50 mg/kg ATRA group (Figure 6D).

**Figure 7:**
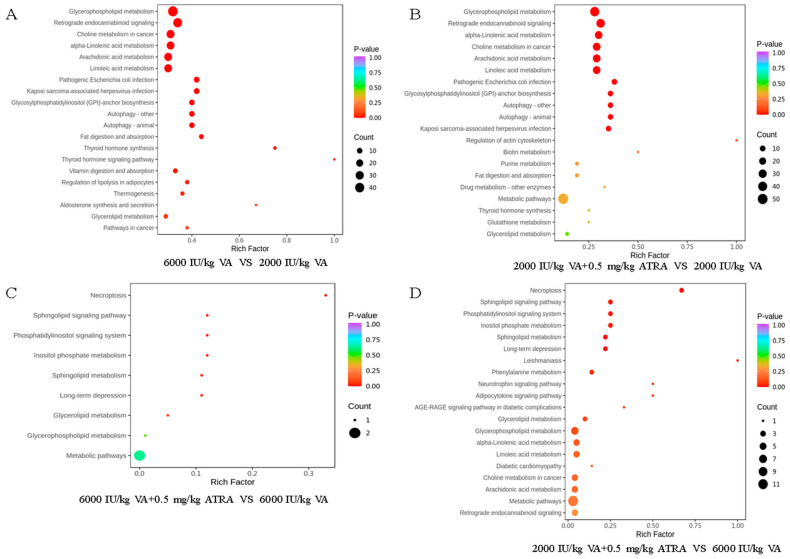
KEGG pathway enrichment analysis. (**A**) KEGG enrichment analysis shown by bubble diagram in the comparison 6000 IU/kg VA vs. 2000 IU/kg VA groups. (**B**) KEGG enrichment analysis shown by bubble diagram in the comparison 2000 IU/kg VA plus 0.5 mg/kg ATRA vs. 2000 IU/kg VA groups. (**C**) KEGG enrichment analysis shown by bubble diagram in the comparison 6000 IU/kg VA plus 0.5 mg/kg ATRA vs. 6000 IU/kg VA groups. (**D**) KEGG enrichment analysis shown by bubble diagram in the comparison 2000 IU/kg VA plus 0.5 mg/kg ATRA vs. 6000 IU/kg VA groups. The color of the point is the *p* values, and the size of the bubble represents the number of metabolites.

KEGG pathway enrichment analysis was used to identify the pathways involved by differential metabolites. As shown in Figure 7A, 13 metabolic pathways were enriched in the 6000 IU/kg group compared with the 2000 IU/kg VA group, including glycerophospholipid metabolism, retrograde endocannabinoid signaling, choline metabolism, alpha-linoleic acid metabolism, arachidonic acid metabolism, linoleic acid metabolism, pathogenic *Escherichia coli* infection, etc. When birds were fed 2000 IU/kg VA, addition with 0.5 mg/kg ATRA significantly impacted the top 7 pathways similar to those previously described (Figure 7B). Furthermore, the necroptosis, sphingolipid signaling pathway, phosphatidylinositol signaling system, inositol phosphate metabolism, sphingolipid metabolism, long-time depression, etc. were significantly enriched in the 6000 IU/kg VA plus 0.5 mg/kg ATRA group and the 2000 IU/kg VA plus 0.5 mg/kg ATRA group, compared with the 6000 IU/kg VA group (Figure 7C,D).

## 4. Discussion

This study demonstrated that dietary supplementation with VA and its biological active metabolite ATRA improved growth performance and intestinal health of broiler chickens. Broilers receiving higher levels of ATRA and VA exhibited the improvements of ADG during starter and grower phases, respectively. The ATRA showed beneficial effects in the early life of broilers [11], and this transient effect underscores the importance of continued posthatch dietary intervention to maintain metabolic benefits. It has been well demonstrated that supplementation of 3000–13,500 IU/kg of VA promoted growth performance of poultry [1,8,23,24,25]. These beneficial outcomes may be mechanistically linked to improvements in intestinal barrier integrity, lifespan of intestinal epithelial cells, and metabolism regulation [26,27].

Intestinal development greatly contributed to poultry production efficiency, which was usually evaluated by the histology morphology [28]. In the present study, VA × ATRA interaction was detected for duodenal VH/CD, ileal VH, and CD in broilers on day 21. Broilers receiving 2000 IU/kg VA or 0.25 mg/kg ATRA had lower duodenal VH/CD and ileal VH. Addition of ATRA led to higher ileal VH and VH/CD and lower ileal CD in broiler chickens on day 35. These results suggested that VA, ATRA, and particularly their combination positively influenced intestinal development by enhancing villus growth and reducing crypt hyperplasia, thereby potentially enhancing nutrient absorption capacity. He et al. (2024) [29] reported that ATRA increased duodenal VH in a dose-dependent way, 5000 and 7500 IU/kg ATRA significantly improved duodenal morphology of cage-stressed young laying ducks fed 5000 IU/kg of VA. They also reported that ATRA promoted cell proliferation and growth in chicken blastoderm cells [30]. Additionally, ATRA attenuated intestinal epithelial cell apoptosis caused by transmissible gastroenteritis virus [31]. Conversely, VA deficiency reduced villus height and influenced the processes of proliferation and maturation of enterocytes in chicks [32], further highlighting its protective role in intestinal health

The integrity of intestinal barrier depends on the barrier structure and function of microbial, chemical, physical, and immune. Tight junctions are mainly composed of integral transmembranes (occludin and claudins) and peripheral membranes (ZO-1) and have beneficial effects on intestinal barrier protection [33,34]. Additionally, members of the mucin family, such as mucin2 and mucin5ac, major components of protective mucus layer, play a key role in the mucosal immune system [35]. Importantly, VA deficiency (0 IU/kg) and inadequacy (1500 IU/kg) can impair *Mucin2* and *Mucin5ac* expression and suppress mucosal immune function [36]. In this study, the highest mRNA expression of Mucin5ac in the jejunal mucosa was presented in the group with combined addition of 6000 IU/kg VA and 0.5 mg/kg ATRA on day 21. Dietary addition of 2000 IU/kg of VA and 0.5 mg/kg ATRA increased the mRNA expression of *Occludin* and *Claudin-1*. The beneficial effects of VA on gene expression of tight junctions in broiler chickens and weaned piglets has been demonstrated [7,37]. The synergistic effects of VA and ATRA on enhancing intestinal barrier integrity may not only improve nutrient absorption and growth performance but also provide better resistance against intestinal pathogens and inflammatory challenges, offering potential applications in optimizing poultry health under stress conditions.

Intestinal stem cells are essential for the continuous renewal and repair of the intestinal epithelium, which is vital for maintaining nutrient absorption and gut integrity [38]. Core intestinal stem cell markers, such as Lgr5+, Znrf3, Olfm4, and TACSTD2, regulate proliferation, differentiation, and stem cell niche maintenance [39]. Among them, Lgr5+ is a well-established marker of active crypt stem cells, while Znrf3 and Olfm4 contribute to niche signaling, and TACSTD2 is associated with epithelial regeneration [40]. In this study, high-dose VA (6000 IU/kg) and ATRA (0.50 mg/kg) significantly up-regulated these markers at day 21, suggesting enhanced stem cell activity and mucosal repair. These findings are consistent with previous studies showing that ATRA promotes crypt base cell proliferation in mice [41] and that VA deficiency impairs epithelial renewal and barrier function [42]. Notably, the combination of 2000 IU/kg VA and 0.50 mg/kg ATRA yielded the highest expression of Znrf3 and SPP1, supporting a synergistic effect. The Znrf3 regulates Wnt signaling, critical for stem cell maintenance [43], while SPP1 (osteopontin) facilitates cell adhesion and tissue remodeling [44]. These results suggested that combined VA and ATRA supplementation might more effectively promote intestinal regeneration through coordinated activation of stem cell-related pathways.

The regulation of retinoic acid homeostasis is critical for maintaining its physiological functions, with key enzymes such as RALDH and CYP26A1 playing central roles in its synthesis and catabolism [45,46]. The RALDH2 catalyzes the conversion of retinal to ATRA, while CYP26A1 ensures retinoic acid catabolism to prevent accumulation [47,48]. In the current study, the combined supplementation with 6000 IU/kg VA and 0.50 mg/kg ATRA had the highest abundance of *RDH10* and *CYP26A1* in broilers aged 21 and 35 days. Addition of 0.50 mg/kg ATRA up-regulated mRNA levels of *RALDH2* in the jejunum of birds on days 21 and 35. These data suggested that the supplementation of VA and ATRA enhanced the synthesis and catabolism of retinoic acid to maintain the homeostasis of retinoic acid.

The heterodimeric nuclear receptor of RARs (RARα, RARβ, and RARγ) and RXRs (RXRα, RXRβ, and RXRγ) is crucial for regulating gene expression [49,50]. Meanwhile, retinoic acid including ATRA exerted protective effects on intestinal health by activating the RAR and RXR family [51,52]. In the present study, VA × ATRA interactions on the mRNA abundance of *RARα*, *RARβ* (on days 21 and 35), and *RXRγ* (on day 21) were observed. The highest abundance levels of *RARα*, *RARβ*, and *RXRγ* were presented in the broilers fed 6000 IU/kg VA plus 0.5 mg/kg ATRA. Dietary addition of 2000 IU/kg VA and 0.5 mg/kg ATRA had the highest abundance of RARβ in birds at 35 days. These findings indicated that dietary VA and ATRA modulated the expression of key nuclear receptors in a dose- and time-dependent manner, potentially enhancing intestinal integrity via RAR/RXR signaling pathways.

The metabolomic analysis further revealed the changes in metabolites and connected pathways in chickens with dietary treatments of VA or ATRA. There were significant changes in plasma metabolism in different levels of dietary VA, which was related to lipid metabolism, pathogenic *Escherichia coli* infection, and vitamin digestion and absorption. The addition of 0.50 mg/kg ATRA greatly affected plasma in birds fed 2000 IU/kg VA rather than 6000 IU/kg, with differentially regulated metabolites involved in retrograde endocannabinoid signaling, lipid metabolism, and pathogenic *Escherichia coli* infection. The pathway of pathogenic *Escherichia coli* infection is associated with the intestinal injury and inflammatory response [53]. A previous study showed that VA could affect the lipid metabolism in the chicks [54]. The ATRA enhanced the transdifferentiation of avian primary myoblasts to adipocytes [55]. In addition, ATRA promoted adipose deposition with hypertrophy in quail and chick embryos [29,56]. The 2000 IU/kg VA plus 0.5 mg/kg ATRA group did not show greatly different plasma metabolomics from the 6000 IU/kg VA group, with 24 different metabolites involved in the sphingolipid signaling pathway, adipocytokine signaling pathway, glycerolipid metabolism, and lipid metabolism. The modulation of lipid metabolism and intestinal integrity by VA and ATRA might be mediated through direct regulation of gene expression, indirect regulation of gut microbiota, and interaction of key metabolic and immune pathways [57,58], which needed further investigation.

## 5. Conclusions

The 0.50 mg/kg ATRA and 6000 IU/kg VA improved the growth performance of broilers in starter and grower phases, respectively. They synergistically improved intestinal integrity, vitamin A metabolism, and plasma lipid metabolism via the activation of retinoic acid receptors. The comparison of their effects with the commercial VA level (12,000 IU/kg) for broilers should be investigated in the future.

## Figures and Tables

**Table 1:** Basal diet composition and nutrient levels (dry matter basis).

Ingredients	Contents (%)	
	Starter (Days 1–21)	Grower (Days 22–35)
Maize	51.90	61.11
Soybean meal	39.80	31.15
Soybean oil	4.20	4.50
Dicalcium phosphate	1.80	1.30
Limestone	1.16	0.83
Sodium chloride	0.20	0.20
*L*-Lysine	0.23	0.23
*D, L*-Methionine	0.19	0.17
Threonine	0.13	0.12
Choline chloride (50%)	0.15	0.15
Mineral premix ^1^	0.20	0.20
Vitamin premix ^2^	0.04	0.04
Total	100.00	100.00
Calculated nutrients ^3^		
Metabolizable energy, MJ/kg	12.38	12.89
Crude protein, %	21.50	18.50
Calcium, %	0.95	0.70
Available phosphorus, %	0.45	0.35
Lysine, %	1.19	1.01
Methionine, %	0.48	0.43
Threonine, %	0.83	0.79

^1^ Mineral premix provided per kilogram of complete diet: Mn, 100 mg; Zn, 100 mg; Fe, 80 mg; Cu, 10 mg; I, 0.7 mg; Se, 0.3 mg. ^2^ Vitamin premix provided per kilogram of complete diet: VD3, 3000 IU; VK3, 3.2 mg; VB1, 3 mg; VB2, 4 mg; VB12, 0.025 mg; VE, 44 IU; biotin, 0.0325 mg; folic acid, 2 mg; pantothenic acid, 15 mg; nicotinic acid, 15 mg. ^3^ Calculated values from Tables of Feed Composition and Nutritive Values in China (version 33, 2022) [16].

**Table 2:** Sequences of primers used for qRT-PCR.

Gene Names	NCBI Number	Forward Sequence (5′-3′)	Reverse Sequence (5′-3′)
*ZO-1*	XM_040706827.2	CTTCAGGGTGTTTCTCTTCCTCCTC	CTGTGGTTTCATGGCTGGATC
*Occludin*	NM_205128.1	ACGGCAGCACCTACCTCAA	GGGCGAAGAAGCAGATGAG
*Claudin-1*	NM_001013611.2	CATACTCCTGGGTCTGGTTGGT	GACAGCCATCCGCATCTTCT
*Mucin2*	XM_040701656.2	TTCATGATGCCTGCTCTTGTG	CCTGAGCCTTGGTACATTCTTGT
*Mucin5ac*	XM_040701669.2	TGTGGTTGCTATGAGAATGGA	TTGCCATGGTTTGTGCAT
*Lgr5^+^*	XM_205518.1	CCTTTATCAGCCCAGAAGTGA	TGGAACAAATGCTACGGATG
*Olfm4*	NM_001040463.1	GACTGGCTCTCTGGATGACC	AGCGTTGTGGCTATCACTTG
*Znrf3*	XM_015275473.1	GCCTCTACCAAGCCCAATCT	GGTCGTCGGAAGTTGTGAG
*TACSTD2*	NM_001277676.2	TGAGAAGCCACCAGTGTTTAG	CTCCGCTGGCACAGAATAAT
*SPP1*	NM_204535.4	GCCCAACATCAGAGCGTAGA	ACGGGTGACCTCGTTGTTTT
*RDH10*	NM001199459.1	AATGCTGGCGTGGTCTC	TCATCGGCTGGTCTGTAAG
*RALDH2*	AF064253	CAAGACATGAACCCATCG	GAGCTGGAGCAATCTTCC
*CYP26A1*	NM001001129.1	TACCGACAAGGACGAGTTCA	CATTGTAGGAGGTCCATTTAGC
*RAR* *α*	NM_204536.1	AGGAGCTGATCGAGAAGG	GAGCTGTTGTTCGTGGTG
*RAR* *β*	NM_205326	GCATCAGTGCAAAAGGTG	TGTCAGTGGTTCGTGTCC
*RAR* *γ*	NM_205294	GATGAAGATCACCGACCTG	TCCTCCTCGAACATCTCG
*RXR* *α*	XM_003642291.6	GATGCGAGACATGCAGATG	GTCGGGGTATTTGTGCTTG
*RXR* *γ*	NM_205294.2	CCAAGACGGAGGCATACAG	GGAGCGATGGGAGAAGGAT
*β-actin*	NM_205518	GAGAAATTGTGCGTGACATCA	CCTGAACCTCTCATTGCCA

Abbreviations: ZO-1, Zonula occludens-1; Lgr5^+^, leucine-rich-repeat-containing G-protein-coupled receptor 5+; Olfm4, Olfactomedin 4; Znrf3, Zinc and ring finger 3; TACSTD2, Tumor associated calcium signal transducer 2; SPP1, secreted phosphoprotein 1; RDH10, retinol dehydrogenase 10; RALDH2, Recombinant aldehyde dehydrogenase 2; CYP26A1, cytochrome P450, family 26, subfamily A, polypeptide 1; RAR, retinoic acid receptor; RXR, retinoic acid X receptor.

**Table 3:** Effects of dietary VA and ATRA supplementation on the growth performance of broiler chickens ^1^.

VA (IU/kg)	2000			6000			SEM	*p* Values		
ATRA (mg/kg)	0	0.25	0.50	0	0.25	0.50		VA	ATRA	VA × ATRA
BW										
Day 1	40.70	40.66	40.61	40.42	40.71	40.89	0.06	0.893	0.469	0.208
Day 21	612.30	647.65	654.16	632.71	631.79	644.37	4.84	0.851	0.071	0.242
Day 35	1474.79	1595.04	1636.89	1670.47	1666.42	1699.63	19.55	0.002	0.202	0.333
Days 1–21										
ADG (g)	26.41	28.69	28.99	27.75	27.72	28.74	0.27	0.935	0.018	0.156
ADFI (g)	40.99	41.44	40.57	40.25	39.76	40.91	0.50	0.518	0.993	0.740
FCR	1.55	1.44	1.40	1.45	1.44	1.42	0.02	0.402	0.105	0.326
Mortality (%)	0	1.39	1.39	6.94	2.78	1.39	0.18	0.055	0.488	0.094
Days 22–35										
ADG (g)	62.77	68.02	70.29	75.52	75.33	75.37	1.37	0.002	0.331	0.329
ADFI (g)	126.31	132.97	131.55	136.96	138.34	132.81	1.09	0.005	0.190	0.144
FCR	2.11	2.00	1.88	1.82	1.84	1.77	0.05	0.057	0.357	0.610
Days 1–35										
ADG (g)	40.95	44.42	45.51	46.86	46.76	47.39	0.58	0.001	0.105	0.189
ADFI (g)	75.11	78.05	76.96	78.93	79.20	77.67	0.52	0.070	0.395	0.403
FCR	1.86	1.77	1.69	1.69	1.69	1.64	0.02	0.044	0.196	0.560

Abbreviations: VA = vitamin A; ATRA = all-trans retinoic acid; ADG = average daily gain; ADFI = average daily feed intake; FCR = feed conversion ratio; SEM = standard error of the means. ^1^ Each value represents the mean values of 6 pens of 12 birds each during days 1–21 and 6 pens of 10 birds each during days 22–35 (*n* = 6).

**Table 4:** Effects of dietary VA and ATRA supplementation on the intestinal morphology of broiler chickens on day 21 ^1^.

VA (IU/kg)	2000			6000			SEM	*p* Values		
ATRA (mg/kg)	0	0.25	0.50	0	0.25	0.50		VA	ATRA	VA × ATRA
Duodenum										
VH (μm)	1338.32	1216.35	1256.85	1324.06	1276.51	1208.38	24.03	0.986	0.208	0.644
CD (μm)	128.02	142.32	120.90	125.00	113.97	108.49	2.67	0.004	0.056	0.099
VH/CD	10.95 ^a^	8.56 ^b^	10.64 ^a^	10.72 ^a^	11.22 ^a^	11.19 ^a^	0.20	0.006	0.031	0.003
Jejunum										
VH (μm)	706.39	780.42	870.88	849.97	707.74	820.86	23.97	0.883	0.203	0.132
CD (μm)	102.93	104.98	108.82	107.62	98.64	112.56	2.08	0.868	0.220	0.493
VH/CD	6.96	7.43	8.01	7.86	7.06	7.19	0.16	0.754	0.641	0.073
Ileum										
VH (μm)	604.80 ^cd^	623.16 ^bc^	631.55 ^b^	618.31 ^bc^	594.34 ^d^	671.84 ^a^	4.10	0.189	<0.001	<0.001
CD (μm)	96.37 ^ab^	100.24 ^a^	99.03 ^ab^	98.46 ^ab^	95.23 ^b^	99.33 ^a^	0.53	0.394	0.317	0.018
VH/CD	6.28	6.22	6.38	6.28	6.26	6.72	0.04	0.068	0.001	0.106

Abbreviations: VA = vitamin A; ATRA = all-trans retinoic acid; VH = villus height; CD = crypt depth; VH/CD = villus height/crypt depth ratio; SEM = standard error of the mean. ^1^ Each value represents the mean values of 12 birds (*n* = 12). ^a–d^ Means with different superscripts in the same row differ with significant VA × ATRA interactions (*p* < 0.05).

**Table 5:** Interactive effects of dietary VA and ATRA supplementation on the intestinal morphology of broiler chickens on day 35 ^1^.

VA (IU/kg)	2000			6000			SEM	*p* Values		
ATRA (mg/kg)	0	0.25	0.50	0	0.25	0.50		VA	ATRA	VA × ATRA
Duodenum										
VH (μm)	1427.09	1222.44	1395.95	1440.13	1432.53	1412.95	29.97	0.182	0.327	0.302
CD (μm)	153.87	164.04	141.43	149.50	175.26	127.82	5.24	0.826	0.021	0.592
VH/CD	10.85	8.07	10.03	9.76	8.26	11.14	0.22	0.846	<0.001	0.060
Jejunum										
VH (μm)	946.15	916.34	964.52	863.19	989.23	924.34	23.39	0.727	0.676	0.397
CD (μm)	117.52	111.76	111.77	103.92	118.27	94.29	2.80	0.137	0.203	0.170
VH/CD	8.16	8.37	8.66	8.35	8.58	9.87	0.20	0.161	0.082	0.465
Ileum										
VH (μm)	704.19	823.11	725.88	725.15	793.91	643.07	19.51	0.425	0.026	0.537
CD (μm)	110.00	114.26	104.26	114.64	109.89	87.59	2.34	0.205	0.003	0.133
VH/CD	6.41	7.21	7.01	6.38	7.26	7.33	0.14	0.692	0.021	0.863

Abbreviations: VA = vitamin A; ATRA = all-trans retinoic acid; VH = villus height; CD = crypt depth; VH/CD = villus height/crypt depth ratio; SEM = standard error of the mean. ^1^ Each value represents the mean values of 12 birds (*n* = 12).

## Data Availability

The original contributions presented in this study are included in the article. Further inquiries can be directed to the corresponding authors.

## Abbreviations

The following abbreviations are used in this manuscript:

ADFI	Average daily feed intake
ADG	Average daily gain
ATRA	All-trans retinoic acid
CD	Crypt depth
CRABP	Cellular retinoic acid-binding protein
CRBP	Cellular retinol-binding protein
CYP26A1	Cytochrome P450, family 26, subfamily A, polypeptide 1
FABP5	Atty acid-binding protein 5
FCR	Feed conversion ratios
IL	Interleukin
Lgr5+	Leucine-rich-repeat-containing G-protein-coupled receptor 5+
Olfm4	Olfactomedin 4
RALDH	Retinal dehydrogenase
RAR	Retinoic acid receptor
RAREs	Retinoic acid-responsive elements
RBP4	Retinol-binding protein
RDH	Retinol dehydrogenase
RDH10	Retinol dehydrogenase 10
RXR	Retinoid receptor X
SEM	Standard error
SPP1	Secreted phosphoprotein 1
TACSTD2	Tumor associated calcium signal transducer 2
VA	Vitamin A
VH	Villus height
Znrf3	Zinc and ring finger 3
ZO-1	Zonula occludens-1

## References

[1] Savaris V.D.L., Pozza P.C., Polese C., de Vargas J.G., Pavlak M.S.D., Wachholz L., Vieira B.S., Tesser G.L.S., de Oliveira Carvalho P.L., Eyng C. (2024). Performance and Bone Characteristics of Broilers Fed Diets Supplemented with Vitamin a at Different Concentrations. J. Anim. Physiol. Anim. Nutr..

[2] El-Ratel I.T., Amara M.M., Beshara M.M., Basuini M.F.E., Fouda S.F., El-Kholy K.H., Ebeid T.A., Kamal M., Othman S.I., Rudayni H.A. (2024). Effects of Supplemental Vitamin a on Reproduction and Antioxidative Status of Aged Laying Hens, and Growth, Blood Indices and Immunity of Their Offspring. Poult. Sci..

[3] Zhang L., Hou Y., Ma Z., Xie J., Fan J., Jiao Y., Wang F., Han Z., Liu S., Ma D. (2023). Effect of Oral Vitamin a Supplementation on Host Immune Response to Infectious Bronchitis Virus Infection in Specific Pathogen-Free Chicken. Poult. Sci..

[4] Uni Z., Zaiger G., Gal-Garber O., Pines M., Rozenboim I., Reifen R. (2000). Vitamin a Deficiency Interferes with Proliferation and Maturation of Cells in the Chicken Small Intestine. Br. Poult. Sci..

[5] Sy A.M., Kumar S.R., Steinberg J., Garcia-Buitrago M.T., Arosemena Benitez L.R. (2020). Liver Damage due to Hypervitaminosis. ACG Case Rep. J..

[6] Khan R.U., Khan A., Naz S., Ullah Q., Puvača N., Laudadio V., Mazzei D., Seidavi A., Ayasan T., Tufarelli V. (2023). Pros and Cons of Dietary Vitamin A and Its Precursors in Poultry Health and Production: A Comprehensive Review. Antioxidants.

[7] Li P., Liu C., Niu J., Zhang Y., Li C., Zhang Z., Guo S., Ding B. (2022). Effects of Dietary Supplementation with Vitamin a on Antioxidant and Intestinal Barrier Function of Broilers Co-Infected with Coccidia and *Clostridium perfringens*. Animals.

[8] Guo S., He L., Zhang Y., Niu J., Li C., Zhang Z., Li P., Ding B. (2023). Effects of Vitamin a on Immune Responses and Vitamin a Metabolism in Broiler Chickens Challenged with Necrotic Enteritis. Life.

[9] Barbalho S.M., Goulart R.A., Batista G.L.S.A. (2019). Vitamin a and Inflammatory Bowel Diseases: From Cellular Studies and Animal Models to Human Disease. Expert Rev. Gastroenterol. Hepatol..

[10] Balmer J.E., Blomhoff R. (2002). Gene Expression Regulation by Retinoic Acid. J. Lipid Res..

[11] Kim D.H., Lee J., Lee C., Shin B.J., Ryu B.Y., Lee K. (2023). Short Communication: In Ovo Injection of All-Trans Retinoic Acid Causes Adipocyte Hypertrophy in Embryos but Lost Its Effect in Posthatch Chickens. Animal.

[12] Pu J., Chen D., Tian G., He J., Zheng P., Huang Z., Mao X., Yu J., Luo Y., Luo J. (2024). All-Trans Retinoic Acid Alleviates Transmissible Gastroenteritis Virus-Induced Intestinal Inflammation and Barrier Dysfunction in Weaned Piglets. J. Anim. Sci. Biotechnol..

[13] Savaris V.D.L., Souza C., Wachholz L., Broch J., Polese C., Carvalho P.L.O., Pozza P.C., Eyng C., Nunes R.V. (2021). Interactions Between Lipid Source and Vitamin A on Broiler Performance, Blood Parameters, Fat and Protein Deposition Rate, and Bone Development. Poult. Sci..

[14] Davis C.Y., Sell J.L. (1983). Effect of All-Trans Retinol and Retinoic Acid Nutriture on the Immune System of Chicks. J. Nutr..

[15] (2004). Feeding Standard of Chicken.

[16] (2022). Tables of Feed Composition and Nutritive Values in China (Version 33).

[17] Li P., Gao M., Fu J., Yan S., Liu Y., Mahmood T., Lv Z., Guo Y. (2022). Dietary Soya Saponin Improves the Lipid Metabolism and Intestinal Health of Laying Hens. Poult. Sci..

[18] Wei H., Zhang J., Yang M., Li Y., Guo K., Qiao H., Xu R., Liu S., Xu C. (2024). Selection and Validation of Reference Genes for Gene Expression in *Bactericera gobica* Loginova under Different Insecticide Stresses. Int. J. Mol. Sci..

[19] Dai D., Wu S.G., Zhang H.J., Qi G.H., Wang J. (2020). Dynamic Alterations in Early Intestinal Development, Microbiota and Metabolome Induced by in Ovo Feeding of L-Arginine in a Layer Chick Model. J. Anim. Sci. Biotechnol..

[20] Ouattara D.A., Remolue L., Becker J., Perret M., Bunescu A., Hennig K., Biliaut E., Badin A., Giacomini C., Reynier F. (2020). An Integrated Transcriptomics and Metabolomics Study of the Immune Response of Newly Hatched Chicks to the Cytosine-Phosphate-Guanine Oligonucleotide Stimulation. Poult. Sci..

[21] Human Metabolome Database. https://hmdb.ca/.

[22] Kyoto Encyclopedia of Genes and Genomes (KEGG) Databases. http://www.genome.jp/kegg/.

[23] Feng Y.L., Xie M., Tang J., Huang W., Zhang Q., Hou S.S. (2019). Effects of Vitamin A on Growth Performance and Tissue Retinol of Starter White Pekin Ducks. Poult. Sci..

[24] Liang J.R., Xiao X., Yang H.M., Wang Z.Y. (2021). Assessment of Vitamin A Requirement of Gosling in 0–28 D Based on Growth Performance and Bone Indexes. Poult. Sci..

[25] Dewi W.K., Aji B.S.P., Fikri F., Purnomo A., Maslamama S.T., Caliskan H., Purnama M.T.E. (2024). Strategies to Combat Heat Stress in Isa Brown Layer Hens: Unveiling the Roles of Vitamin A, Vitamin E, Vitamin K, Vitamin C, Selenium, Folic Acid, and in Combination. Open Vet. J..

[26] Wang Z., Li J., Wang Y., Wang L., Yin Y., Yin L., Yang H., Yin Y. (2020). Dietary Vitamin A Affects Growth Performance, Intestinal Development, and Functions in Weaned Piglets by Affecting Intestinal Stem Cells. J. Anim. Sci..

[27] Zhou J., Qin Y., Xiong X., Wang Z., Wang M., Wang Y., Wang Q.Y., Yang H.S., Yin Y. (2021). Effects of Iron, Vitamin a, and the Interaction between the Two Nutrients on Intestinal Development and Cell Differentiation in Piglets. J. Anim. Sci..

[28] Shastak Y., Pelletier W. (2024). Delving into Vitamin A Supplementation in Poultry Nutrition: Current Knowledge, Functional Effects, and Practical Implications. World Poult. Sci. J..

[29] He X., Wei W., Liu J., Liang Z., Wu Y., Liu J., Pi J., Zhang H. (2024). Whole-Transcriptome Analysis Reveals the Effect of Retinoic Acid on Small Intestinal Mucosal Injury in Cage-Stressed Young Laying Ducks. Poult. Sci..

[30] Lu Y., Wang H., Cao H., Chen X., Li D., Yu D., Yu M. (2022). Ascorbic Acid and All-Trans Retinoic Acid Promote Proliferation of Chicken Blastoderm Cells (Cbcs) by Mediating DNA Demethylation. In Vitro Cell. Dev. Biol. Anim..

[31] Pu J., Chen D., Tian G., He J., Huang Z., Zheng P., Mao X., Yu J., Luo J., Luo Y. (2022). All-Trans Retinoic Acid Attenuates Transmissible Gastroenteritis Virus-Induced Apoptosis in Ipec-J2 Cells Via Inhibiting Ros-Mediated P(38)Mapk Signaling Pathway. Antioxidants.

[32] Uni Z., Zaiger G., Reifen R. (1998). Vitamin a Deficiency Induces Morphometric Changes and Decreased Functionality in Chicken Small Intestine. Br. J. Nutr..

[33] Zhang M., Kou J., Wu Y., Wang M., Zhou X., Yang Y., Wu Z. (2020). Dietary Genistein Supplementation Improves Intestinal Mucosal Barrier Function in *Escherichia coli* O78-Challenged Broilers. J. Nutr. Biochem..

[34] Hu Y., Zhang W., Yang K., Lin X., Liu H.C., Odle J., See M.T., Cui X., Li T., Wang S. (2024). Dietary Zn Proteinate with Moderate Chelation Strength Alleviates Heat Stress-Induced Intestinal Barrier Function Damage by Promoting Expression of Tight Junction Proteins Via the A20/Nf-Kappab P65/Mmp-2 Pathway in the Jejunum of Broilers. J. Anim. Sci. Biotechnol..

[35] Gharib-Naseri K., Kheravii S., Keerqin C., Swick R.A., Choct M., Wu S.B. (2021). Differential Expression of Intestinal Genes in Necrotic Enteritis Challenged Broiler Chickens with 2 Different Clostridium Perfringens Strains. Poult. Sci..

[36] Fan X., Liu S., Liu G., Zhao J., Jiao H., Wang X., Song Z., Lin H. (2015). Vitamin a Deficiency Impairs Mucin Expression and Suppresses the Mucosal Immune Function of the Respiratory Tract in Chicks. PLoS ONE.

[37] Wu S., Wang L., Cui B., Wen X., Jiang Z., Hu S. (2023). Effects of Vitamin a on Growth Performance, Antioxidants, Gut Inflammation, and Microbes in Weaned Piglets. Antioxidants.

[38] Barker N. (2014). Adult Intestinal Stem Cells: Critical Drivers of Epithelial Homeostasis and Regeneration. Nat. Rev. Mol. Cell Biol..

[39] Yan K.S., Janda C.Y., Chang J., Zheng G.X.Y., Larkin K.A., Luca V.C., Chia L.A., Mah A.T., Han A., Terry J.M. (2017). Non-Equivalence of Wnt and R-Spondin Ligands During Lgr5(+) Intestinal Stem-Cell Self-Renewal. Nature.

[40] Santos A.J.M., Lo Y.H., Mah A.T., Kuo C.J. (2018). The Intestinal Stem Cell Niche: Homeostasis and Adaptations. Trends Cell Biol..

[41] Conway T.F., Hammer L., Furtado S., Mathiowitz E., Nicoletti F., Mangano K., Egilmez N.K., Auci D.L. (2015). Oral Delivery of Particulate Transforming Growth Factor Beta 1 and All-Trans Retinoic Acid Reduces Gut Inflammation in Murine Models of Inflammatory Bowel Disease. J. Crohns Colitis.

[42] McCullough F.S., Northrop-Clewes C.A., Thurnham D.I. (1999). The Effect of Vitamin a on Epithelial Integrity. Proc. Nutr. Soc..

[43] Koo B.K., Spit M., Jordens I., Low T.Y., Stange D.E., van de Wetering M., van Es J.H., Mohammed S., Heck A.J., Maurice M.M. (2012). Tumour Suppressor Rnf43 Is a Stem-Cell E3 Ligase That Induces Endocytosis of Wnt Receptors. Nature.

[44] Li C., Sun Y., He T., Lu Y., Szeto I.M., Duan S., Zhang Y., Liu B., Zhang Y., Zhang W. (2023). Synergistic Effect of Lactoferrin and Osteopontin on Intestinal Barrier Injury. Int. J. Biol. Macromol..

[45] Yabut K.C.B., Isoherranen N. (2022). Crabps Alter All-Trans-Retinoic Acid Metabolism by Cyp26a1 Via Protein-Protein Interactions. Nutrients.

[46] Yoo H.S., Cockrum M.A., Napoli J.L. (2023). Cyp26a1 Supports Postnatal Retinoic Acid Homeostasis and Glucoregulatory Control. J. Biol. Chem..

[47] Berggren K., McCaffery P., Drager U., Forehand C.J. (1999). Differential Distribution of Retinoic Acid Synthesis in the Chicken Embryo as Determined by Immunolocalization of the Retinoic Acid Synthetic Enzyme, RALDH-2. Dev. Biol..

[48] Yu M., Yu P., Leghari I.H., Ge C., Mi Y., Zhang C. (2013). Raldh2, the Enzyme for Retinoic Acid Synthesis, Mediates Meiosis Initiation in Germ Cells of the Female Embryonic Chickens. Amino Acids.

[49] Li D., Zimmerman T.L., Thevananther S., Lee H.Y., Kurie J.M., Karpen S.J. (2002). Interleukin-1 Beta-Mediated Suppression of Rxr:Rar Transactivation of the Ntcp Promoter Is Jnk-Dependent. J. Biol. Chem..

[50] van Neerven S., Kampmann E., Mey J. (2008). Rar/Rxr and Ppar/Rxr Signaling in Neurological and Psychiatric Diseases. Prog. Neurobiol..

[51] Iwata M., Hirakiyama A., Eshima Y., Kagechika H., Kato C., Song S.Y. (2004). Retinoic Acid Imprints Gut-Homing Specificity on T Cells. Immunity.

[52] Takeuchi H., Yokota A., Ohoka Y., Kagechika H., Kato C., Song S.Y., Iwata M. (2010). Efficient Induction of Ccr9 on T Cells Requires Coactivation of Retinoic Acid Receptors and Retinoid X Receptors (Rxrs): Exaggerated T Cell Homing to the Intestine by Rxr Activation with Organotins. J. Immunol..

[53] Farouk S.M., Abdel-Rahman H.G., Abdallah O.A., El-Behidy N.G. (2022). Comparative Immunomodulatory Efficacy of Rosemary and Fenugreek against *Escherichia coli* Infection Via Suppression of Inflammation and Oxidative Stress in Broilers. Environ. Sci. Pollut. Res. Int..

[54] Sklan D. (1983). Effect of High Vitamin a or Tocopherol Intake on Hepatic Lipid Metabolism and Intestinal Absorption and Secretion of Lipids and Bile Acids in the Chick. Br. J. Nutr..

[55] Kim D.H., Lee J., Suh Y., Ko J.K., Lee K. (2022). Transdifferentiation of Myoblasts into Adipocytes by All-Trans-Retinoic Acid in Avian. Front. Cell Dev. Biol..

[56] Kim D.H., Lee J., Kim S., Lillehoj H.S., Lee K. (2021). Hypertrophy of Adipose Tissues in Quail Embryos by in Ovo Injection of All-Trans Retinoic Acid. Front. Physiol..

[57] Pan P., Atkinson S.N., Taylor B., Zhu H., Zhou D., Flejsierowicz P., Wang L.S., Morse M., Liu C., Gunsolus I.L. (2021). Retinoic Acid Signaling Modulates Recipient Gut Barrier Integrity and Microbiota After Allogeneic Hematopoietic Stem Cell Transplantation in Mice. Front. Immunol..

[58] Zhang X., Xu H., Zhang C., Bai J., Song J., Hao B., Zhang L., Xia G. (2022). Effects of Vitamin A on Yanbian Yellow Cattle and Their Preadipocytes by Activating AKT/mTOR Signaling Pathway and Intestinal Microflora. Animals.

